# Induction of systemic, mucosal, and cellular immunity against SARS‐CoV‐2 in mice vaccinated by trans‐airway with a S1 protein combined with a pulmonary surfactant‐derived adjuvant SF‐10

**DOI:** 10.1111/irv.13119

**Published:** 2023-03-09

**Authors:** Takashi Kimoto, Satoko Sakai, Keiko Kameda, Ryoko Morita, Etsuhisa Takahashi, Yasuo Shinohara, Hiroshi Kido

**Affiliations:** ^1^ Division of Enzyme Chemistry, Institute for Enzyme Research Tokushima University Tokushima Japan; ^2^ Faculty of Pharmaceutical Sciences Tokushima University Tokushima Japan; ^3^ Institute for Genome Research Tokushima University Tokushima Japan

**Keywords:** mucosal adjuvant, pulmonary surfactant, respiratory mucosal immunity, SARS‐CoV‐2 vaccine, trans‐airway route

## Abstract

**Background:**

There is a need for vaccines that can induce effective systemic, respiratory mucosal, and cellular immunity to control the COVID‐19 pandemic. We reported previously that a synthetic mucosal adjuvant SF‐10 derived from human pulmonary surfactant works as an efficient antigen delivery vehicle to antigen presenting cells in the respiratory and gastrointestinal tracts and promotes induction of influenza virus antigen‐specific serum IgG, mucosal IgA, and cellular immunity.

**Methods:**

The aim of the present study was to determine the effectiveness of a new administration route of trans‐airway (TA) vaccine comprising recombinant SARS‐CoV‐2 spike protein 1 (S1) combined with SF‐10 (S1‐SF‐10 vaccine) on systemic, local, and cellular immunity in mice, compared with intramuscular injection (IM) of S1 with a potent adjuvant AddaS03™ (S1‐AddaS03™ vaccine).

**Results:**

S1‐SF‐10‐TA vaccine induced S1‐specific IgG and IgA in serum and lung mucosae. These IgG and IgA induced by S1‐SF‐10‐TA showed significant protective immunity in a receptor binding inhibition test of S1 and angiotensin converting enzyme 2, a receptor of SARS‐CoV‐2, which were more potent and faster achievement than S1‐AddaS03™‐IM. Enzyme‐linked immunospot assay showed high numbers of S1‐specific IgA and IgG secreting cells (ASCs) and S1‐responsive IFN‐γ, IL‐4, IL‐17A cytokine secreting cells (CSCs) in the spleen and lungs. S1‐AddaS03™‐IM induced IgG ASCs and IL‐4 CSCs in spleen higher than S1‐SF‐10‐TA, but the numbers of ASCs and CSCs in lungs were low and hardly detected.

**Conclusions:**

Based on the need for effective systemic, respiratory, and cellular immunity, the S1‐SF‐10‐TA vaccine seems promising mucosal vaccine against respiratory infection of SARS‐CoV‐2.

## 1 INTRODUCTION

Severe acute respiratory syndrome coronavirus 2 (SARS‐CoV‐2) appeared in Wuhan, China, in late of 2019, causing coronavirus disease 2019 (COVID‐19) and infecting approximately 755 million individuals worldwide based on January 2023 data, and death of approximately 6.8 million individuals.^1^ The seriousness of the global situation called for urgent development of COVID‐19 vaccine, and several vaccines were approved at unprecedented speed and used clinically throughout the world.^2^, ^3^, ^4^

The majority of approved vaccines for which clinical trial data disclosed are delivered by intramuscular (IM) injection and induce SARS‐CoV‐2 specific IgG in serum and showed effectiveness in prevention of severe infection and death.^5^ However, vaccines administrated intramuscularly are, in general, known to be minimally effective in inducing mucosal secretory IgA (S‐IgA), which plays an important role in boosting mucosal immunity, and preventing respiratory and gastrointestinal tract infections and viral transmission.^6^ Recent reports have described IM administration of COVID‐19 mRNA vaccine (e.g., Comirnaty)‐induced SARS‐CoV‐2 specific IgA at mucosal sites.^7^, ^8^ However, the effect of mucosal IgA induced by mRNA vaccine is presumably limited in providing protection against virus infection through mucosal sites.^9^ Thus, although the first‐generation COVID‐19 vaccines produced public benefits, such as reduction of severe infection and death in pandemic emergency situation, it is necessary to develop the next generation COVID‐19 vaccines that can induce more effective mucosal S‐IgA, in addition to systemic IgG and IgA, to achieve protection against infection and prevention of transmission of the virus.

Mucosal vaccines that are directly administered onto mucosal surfaces, such as the nasal cavity and gastrointestinal tract, can induce mucosal S‐IgA.^10^ Since the mucosa is equipped with exclusion systems, namely, the cilia, mucus, and various proteases, against foreign substances including vaccine antigens,^11^ the use of vaccines that contain antigen alone is unlikely to produce protective immunity. Thus, for successful mucosal vaccination, live virus vaccines and mucosal adjuvants that enhance the vaccine effects have been developed worldwide.^12^ However, the live attenuated vaccines, such as Flumist®, have problems related to their efficacy and safety, and thus, their worldwide use is limited.^13^

To overcome these problems, we reported previously the use of an antigen delivery type adjuvant,^14^ pulmonary surfactant (PS)‐based compound, Surfacten®, as an effective and safe intranasal mucosal adjuvant for influenza ether‐split hemagglutinin vaccine (HAv) and that it enhanced the production of both HAv‐specific IgG in serum and S‐IgA in the respiratory mucosae of mice and swine.^15^, ^16^ Based on these results, we then successfully developed synthetic surfactant (SSF), which mimicked human PS, consisting of three major lipids (1,2‐dipalmitoyl‐phosphatidylcholine [DPPC], phosphatidylglycerol [PG], and palmitic acid [PA]) plus the K6L16 peptide (which resembles surfactant protein C [SP‐C]), as a potent and safe synthetic mucosal adjuvant.^17^, ^18^ In addition, to increase the effectiveness of the vaccine, we prepared the antigen and SSF complex by lyophilization, thus allowing the lyophilized powder to be suspended in carboxy vinyl polymer (CVP) to increase the viscosity of the antigen and SSF complex solution in order to prolong antigen uptake on the mucosal surface.^18^ The final vaccine solution was termed antigen‐SF‐10. In a series of studies, we reported that intranasal application of antigen‐SF‐10 enhanced the absorption of antigens to nasal antigen presenting cells and induced systemic as well as respiratory humoral immunity in mice and cynomolgus monkeys^18^, ^19^, ^20^ and cell‐mediated immunity in mice.^21^

The present study is an extension to the above work and was designed to determine the outcome of mucosal application of recombinant S1 spike protein of SARS‐CoV‐2, as an antigen combined with SF‐10 (S1‐SF‐10) on respiratory and systemic immunity against SARS‐CoV‐2 infection. For this purpose, we developed S1‐SF‐10 trans‐airway (TA) vaccination, which was inoculated through the nasal cavity and reached the lower respiratory tract of mice, the site of viral infection and proliferation. To test the effectiveness of a new administration method of TA vaccination, we analyzed the levels of induced S1‐specific antibodies, the antibody secreting cells (ASCs), and S1‐responsive Th cytokine secreting cells (CSCs) in lung lymphocytes and splenocytes. We also compared the level of protective immunity induced by S1‐SF‐10‐TA and IM injection of S1 with an already reported potent adjuvant AddaS03™, like AS03 adjuvant, (S1‐AddaS03™‐IM) against SARS‐CoV‐2 infection using the S1/angiotensin converting enzyme 2 (ACE2) binding inhibition (BI) assay.

## 2 METHODS

### 2.1 Antigen and animals

HEK293 expressing recombinant SARS‐CoV‐2 S1 of the original Wuhan strain (Assession# QHD43416.1, Val 16–Arg 685) was purchased from ACROBiosystems (Newark, DE). Female BALB/c mice (age 6–8 weeks) were purchased from Japan SLC (Shizuoka, Japan). All animals were maintained under specific‐pathogen‐free conditions. Mice were anesthetized by intraperitoneal injection of 62.6 mg ketamine and 12.4 mg xylazine per kg body weight before vaccination.

### 2.2 Preparation of S1‐SF‐10

The procedure used for the preparation of SSF was described in detail previously.^14^, ^17^ Briefly, SSF was prepared by mixing DPPC, PG, and PA, and K6L16 peptide at a weight ratio of 75:25:10:2. SSF was mixed with S1 at a SSF phospholipid: S1 protein ratio of 10:1 at 42°C, and the S1‐SSF complex was prepared by lyophilization and stored at −20°C until use. To prepare 0.1% CVP solution, CVP powder (Carbopol®971P NF POLYMER, Lubrizol, Cleveland, OH) was dissolved in saline (pH 7.0–7.5) by stirring, followed by homogenizer and then stored at 4°C until use. Before administration, the lyophilized S1‐SSF was dissolved in 0.1% CVP solution.

### 2.3 Immunization and sampling of serum and BALF

Mice were vaccinated by TA route with 1 or 10 μg of S1 antigen with or without SF‐10 twice or thrice every 2 weeks. TA vaccination was performed by administering 30 μL of S1‐SF‐10 or saline containing S1 antigen into the nasal cavity of mice using a pipette; the dosage is the amount sufficient covering a mucous membrane from the nasal cavity to the lower respiratory tract (Figure S1).

As a positive control group, mice were immunized intramuscularly (into thigh muscles) with 10 μg S1 in 50 μL of saline or 10 μg S1 mixed with an adjuvant AddaS03™ (InvivoGen, San Diego, CA) using a 1 mL plastic syringe. S1‐AddaS03™ solution was prepared according to the instructions provided by the manufacturer. At 2 weeks after the last immunization, serum and bronchoalveolar lavage fluid (BALF) samples were obtained as described in detail previously.^15^

### 2.4 Measurement of S1‐specific IgA, S‐IgA, and IgG

Two weeks after the last immunization, we measured S1‐specific IgA and IgG levels in the serum and S1‐specific S‐IgA and S‐IgG levels in BALF by enzyme‐linked immunosorbent assay (ELISA). A 96‐well plate (Nunc, Naperville, IL) was coated with S1 (0.1 μg/well) in ELISA Coating Buffer (Bethyl Laboratories, Montgomery, TX) overnight at 4°C, then blocked with Tris buffered saline with BSA (pH 8.0) (TBS, Sigma‐Aldrich, St. Louis, MO) for 2 h at 37°C. Serum and BALF were added to the plate and twofold serially diluted with 0.05% Tween 20‐TBS. The plate was washed six times with 50 mM Tris–HCl (pH 8.0) containing 0.14 M NaCl and 0.05% Tween 20 (TTS), incubated with goat anti‐mouse IgA or anti‐mouse IgG Ab conjugated with HRP (Sigma‐Aldrich) for 2 h at 37°C. The plate was washed six times with TTS and incubated with a KPL TMB Microwell Peroxidase Substrate System (SeraCare, Milford, MA) according to the instructions supplied by the manufacturer. The produced chromogen was measured at 450 nm absorbance using a SpectraMax ABS PLUS (Molecular Devices, Sunnyvale, CA). Antibody titers are defined as the reciprocal of the highest dilution of the sample with optical density (OD) of >0.1.

### 2.5 SARS‐CoV‐2 spike protein 1 and ACE2 BI by sera and BALF of immunized mice

Two weeks after the last immunization, we analyzed inhibition ability of Spike: ACE2 binding in serum and BALF by using Spike S1 (SARS‐CoV‐2): ACE2 Inhibitor Screening Colorimetric Assay Kit (BPS Bioscience, San Diego, CA) according to the instructions provided by the manufacturer. Briefly, the serum and BALF samples were first added to the 96‐well plate (Nunc) coated with Spike S1 then diluted 2‐fold serially at 1/100 to 1/3200 and 1/2 to 1/64, respectively. Subsequently, ACE2‐Biotin, Streptavidin‐HRP, and TMB were reacted in the 96‐well plate, respectively. The chromogen produced was measured at an absorbance of 450 nm using a SpectraMax ABS PLUS. BI (%) was obtained from the following formula: binding inhibition (%) = 100 × (([OD value in the absence of specimen] − [OD value in the presence of specimen]) ÷ [OD value in the absence of specimen]).

### 2.6 Detection of S1‐specific antibody and S1‐responsive Th CSCs in spleen and lungs by ELISPOT assay

Two weeks after triple vaccination, the spleen and lungs were dissected out carefully from the control non‐treated mice and immunized mice. Lymphocytes were isolated from the spleen using the method described in detail previously,^21^ whereas lung lymphocytes were isolated by collagenase digestion followed by Percoll (GE Healthcare, Buckinghamshire, UK) density gradient centrifugation, as described previously.^21^

S1‐specific ASCs were detected by enzyme‐linked immunospot (ELISPOT) assay as described previously.^19^ Briefly, the isolated lymphocytes of lung and spleen were seeded at 2 × 10^5^ cells (lung) or 1 × 10^6^ cells (spleen)/well onto MultiScreen 96‐well plates MSIPS4510 (Millipore, Bedford, MA) that had been coated with 1 μg S1 protein/well and incubated with fresh complete RPMI (cRPMI) medium (containing 10 mM HEPES, pH 7.2, 1 mM sodium pyruvate, 1% MEM non‐essential amino acids solution, 14.3 μM 2‐mercaptoethanol, 10 μg/mL gentamycin and 10% heat‐inactivated fetal bovine serum) for 4 h (lung lymphocytes) or 24 h (splenocytes). After incubation, the S1‐specific ASCs spots were detected by using goat anti‐mouse IgG or anti‐mouse IgA antibody conjugated with horseradish peroxidase (Sigma‐Aldrich) and 3‐amino‐9‐ethylcarbazole (Sigma‐Aldrich). The number of ASC spots was counted by ELISPOT counter ImmunoSpot S6 (CTL, Cleveland, OH).

The S1‐responsive Th CSCs were detected by the ELISPOT assay using Mouse IFN‐γ, IL‐4, and IL‐17A ELISpot BASIC kit (HRP) (MABTECH, Nacka Strand, Sweden), according to the protocols provided by the manufacturers. The splenocytes and lung lymphocytes were seeded at 1 × 10^5^ cells (lung) or 1 × 10^6^ cells (spleen)/well onto MultiScreen 96‐well plates MSIPS4510 that had been coated with each cytokine captor antibody and incubated with fresh cRPMI for 24 h with or without 20 μg/mL S1 protein. After incubation, the S1‐responsive Th CSC spots were detected by each biotin conjugated cytokine detection antibody, streptavidin‐horseradish peroxidase and 3‐amino‐9‐ethylcarbazole. The number of CSC spots was counted by ELISPOT counter ImmunoSpot S6.

### 2.7 Statistical analysis

Differences in parameters between the experimental groups were analyzed by the Mann–Whitney *U*‐test between each two groups using JMP® 14 software (SAS Institute Inc., Cary, NC).

## 3 RESULTS

### 3.1 TA administration of S1‐SF‐10 vaccine induces S1‐specific humoral immunities in sera and respiratory mucosal fluid

**FIGURE 1:**
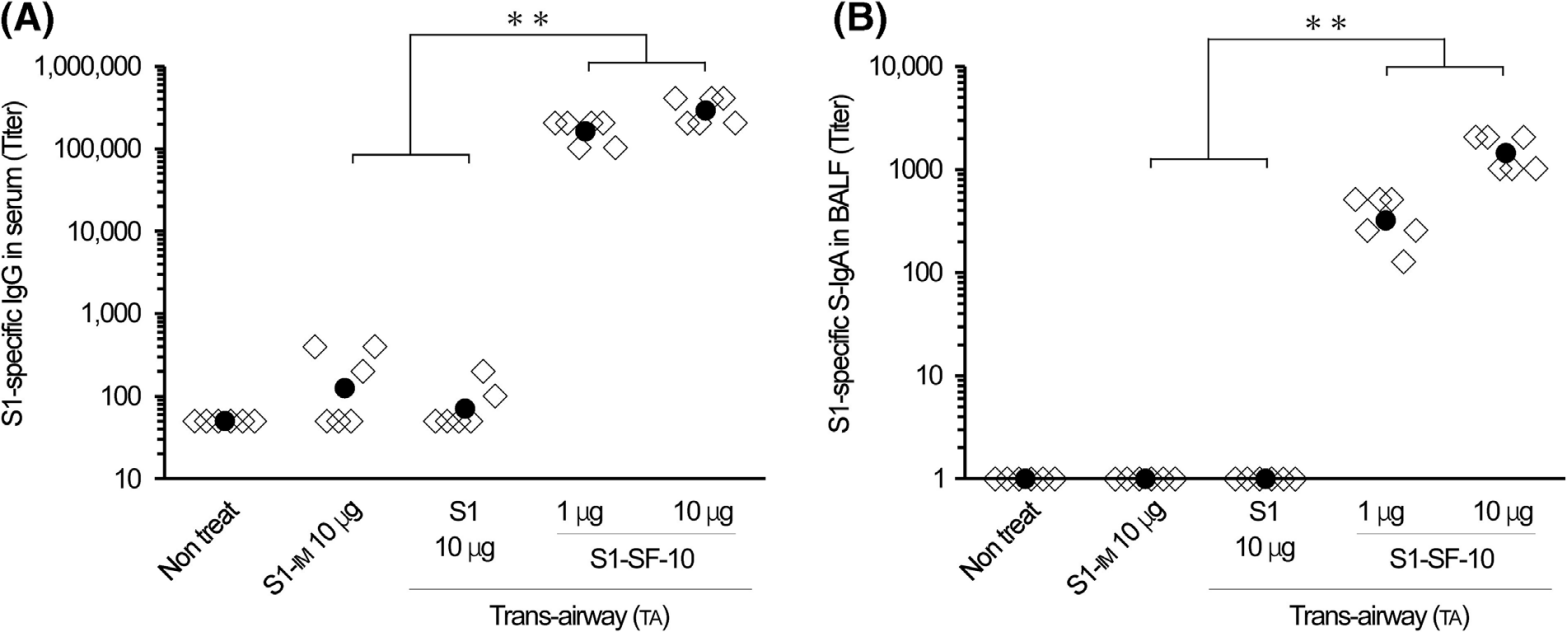
Detection of S1‐specific IgG and IgA antibodies in serum and bronchoalveolar lavage fluid (BALF). Mice were immunized twice by trans‐airway (TA) route administration of S1 combined with or without SF‐10 at Days 0 and 14. Another group of mice were immunized by intramuscular (IM) injection of S1 at the same time schedules. At 2 weeks after the last immunization, the levels of S1‐specific IgG in serum (A) and S‐IgA in BALF (B) were measured by ELISA. Data represent S1‐specific antibody titer of individual mice (open rhombus) and their geometric mean (solid circles) (*n* = 6 mice per group). Differences between same‐time immunization groups were analyzed by the non‐parametric Mann–Whitney *U*‐test. **P < 0.01.

To analyze SF‐10 adjuvanticity against S1 antigen, mice were immunized twice with 10 μg S1 by IM and TA route administration and 1 and 10 μg S1‐SF‐10 vaccine by TA route. At 2 weeks after the last immunization, the S1‐specific IgG in serum and S‐IgA in BALF were measured by ELISA (Figure 1). S1‐IM and S1‐TA antigen induced very low levels of S1‐specific IgG in serum. In contrast, S1‐SF‐10‐TA vaccine induced noticeable levels of S1‐specific IgG in serum at over 100‐fold higher than S1 antigen alone (Figure 1A).

On the other hand, S1‐specific S‐IgA induction in BALF was detected in the 1 and 10 μg S1‐SF‐10‐TA group but none of the S1‐TA and S1‐IM antigen groups (Figure 1B). These results indicate that SF‐10‐TA had immunostimulatory effects as mucosal adjuvant for SARS‐CoV‐2 vaccine.

### 3.2 TA mucosal immunization with S1‐SF‐10 induces rapid and effective protective humoral immunity against SARS‐CoV‐2 compared with IM immunization with S1‐AddaS03™ in S1/ACE2 binding inhibitor screening assay

**FIGURE 2:**
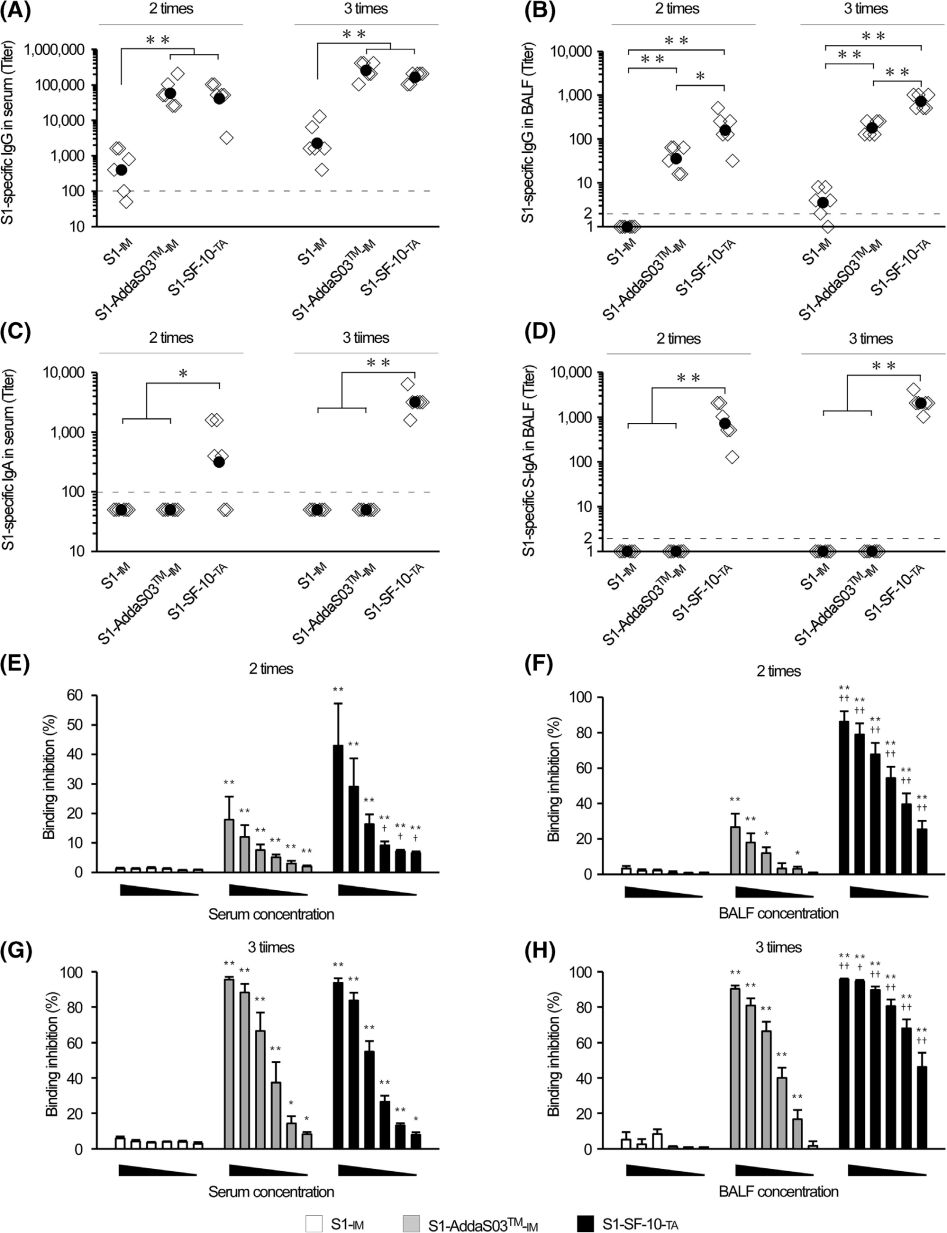
Comparison of the effects of route of vaccine administration on IgG and IgA induction. Mice were immunized twice or thrice by 10 μg of S1‐IM, S1‐AddaS03™‐IM, and S1‐SF‐10‐TA every 2 weeks. At 2 weeks after the last immunization, serum and bronchoalveolar lavage fluid (BALF) samples were collected. ELISA was used to measure the levels of S1‐specific IgG in serum (A), IgG in BALF (B), IgA in serum (C), and S‐IgA in BALF (D). Data represent S1‐specific antibody titer of individual mice (open rhombus) and their geometric mean (solid circles) (*n* = 6 mice per group). Inhibition abilities of Spike/ACE2 binding of serum (E, G) and BALF (F, H) were measured by a colorimetric assay using ACE2 Inhibitor Screening Colorimetric Assay Kit. The serum and BALF samples were diluted serially 2‐fold at 1/100 to 1/3200 and 1/2 to 1/64, respectively. The formula used to calculate the binding inhibition (BI) (%) was described in Materials and Methods. Each bar represents the geometric mean ± SEM of BI (%) of each dilution point samples of S1‐IM (open bars), S1‐AddaS03™‐IM (gray bars), and S1‐SF‐10‐TA (closed bars), (*n* = 6 mice per group). Differences between same‐time immunization groups or same‐serum and BALF dilution groups were analyzed by the non‐parametric Mann–Whitney *U*‐test. *P < 0.05, **P < 0.01.

Next, we compared the effectiveness of S1‐SF‐10‐TA with that of IM S1‐AddaS03™ (a potent IM adjuvant and clinically used candidate of COVID‐19 vaccine adjuvant reported)^22^, ^23^ (Figure 2A–D). The serum levels of S1‐specific IgG were significantly higher in the S1‐AddaS03™‐IM group compared with the S1‐IM group, and similar to those in the S1‐SF‐10‐TA group after double and triple vaccinations (Figure 2A). However, the BALF levels of S1‐specific IgG were significantly lower in the S1‐AddaS03™‐IM group than in the S1‐SF‐10‐TA group (Figure 2B). Serum and BALF S1‐specific IgA were also induced in S1‐SF‐10‐TA group but not S1‐IM group (Figure 2C,D). In addition, S1‐specific antibody levels induced by vaccination were significantly higher than those by double vaccination in serum IgG of S1‐AddaS03™‐IM and S1‐SF‐10‐TA, BALF IgG of S1‐IM, S1‐AddaS03™‐IM and S1‐SF‐10‐TA, and serum IgA of S1‐SF‐10‐TA (*P* < 0.05).

Next, we applied the S1/ACE2 Inhibitor Screening Colorimetric Assay^24^ to determine the protective immunity against SARS‐CoV‐2 of IgG in serum and S‐IgA in BALF induced by S1‐SF‐10‐TA vaccine. The S1:ACE2 BI (%) of serum and BALF of mice immunized with S1‐IM was <10% at any concentration of specimens after double and triple vaccinations (Figure 2E–H). The BI of mice that received double vaccination with S1‐AddaS03™‐IM was approximately 20% at the highest concentration of serum and BALF (Figure 2E,F). In contrast, the BIs of mice immunized double with S1‐SF‐10‐TA were approximately 40% in serum and 80% in BALF at the highest concentration (Figure 2E,F). After triple vaccinations, the BI levels of S1‐AddaS03™‐IM and S1‐SF‐10‐TA were over 80% at the highest concentrations of both serum and BALF (Figure 2G,H). Although the BI values of BALF after triple vaccinations with S1‐SF‐10‐TA were approximately 50% even at the lowest concentrations, those of mice immunized with S1‐AddaS03™‐IM were very low at <2% (Figure 2H). These results indicate that S1‐SF‐10‐TA rapidly and effectively induces protective immunity against SARS‐CoV‐2 systemically in serum and in the respiratory mucosa compared with S1‐AddaS03™‐IM.

### 3.3 TA immunization with S1‐SF‐10 induces S1‐specific ASCs in spleen and lungs

**FIGURE 3:**
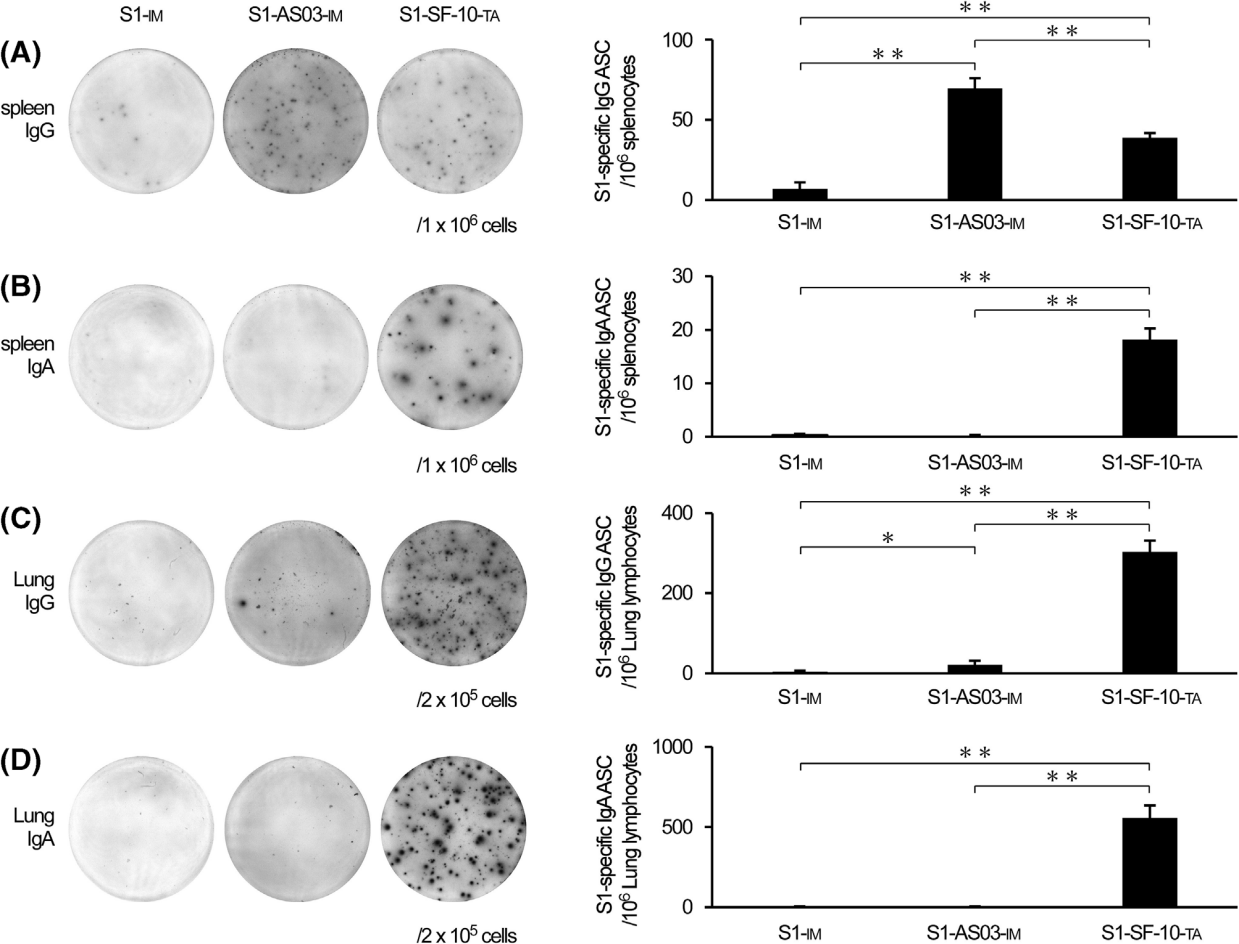
Detection of S1‐specific ASCs in spleen and lung tissues. Mice were triple vaccinated by S1‐IM, S1‐AddaS03™‐IM, and S1‐SF‐10‐TA every 2 weeks. At 2 weeks after the last immunization, the spleen and lungs were dissected out and lymphocytes isolated. The latter were seeded onto S1‐coated plates at 2 × 10^5^ (lungs) or 10^6^ (spleen) cells, and S1‐specific IgG ASCs in spleen (A), IgA ASCs in spleen (B), IgG ASCs in lungs (C), and IgA ASCs in lungs (D) were detected by ELISPOT assay. Each bar represents mean ± SEM counts of ASC per 10^6^ cells (*n* = 10). Differences between groups were analyzed by the non‐parametric Mann–Whitney *U*‐test. *P < 0.05, **P < 0.01.

S1‐SF‐10‐TA resulted in marked induction of both S‐IgA and IgG in BALF (Figure 2B,D). To determine the antibody producing sites, we analyzed S1‐specific IgG and IgA ASCs. For this purpose, lymphocytes were harvested from the spleen and lungs of triple‐vaccinated mice with 10 μg of S1‐IM, S1‐AddaS03™‐IM and S1‐SF‐10‐TA at 2 weeks after the last immunization, and the S1‐specific IgG and IgA ASCs were detected by ELISPOT assay (Figure 3). Only a few S1‐specific IgG ASCs were detected in the spleen of S1‐IM group, and S1‐specific IgA in the spleen, IgG and IgA in the lungs were even below the detection limits. Although the number of S1‐specific IgG ASCs in the spleen of the S1‐AddaS03™‐IM group was higher than approximately 2‐fold of that of S1‐SF‐10‐TA group (Figure 3A), their number was lower in the lungs of mice immunized with S1‐AddaS03™‐IM at approximately 1/15‐fold of that found in S1‐SF‐10‐TA group (Figure 3C). In addition, S1‐specific IgA ASCs in the both spleen and lung were detected in S1‐SF‐10‐TA group but hardly S1‐AddaS03™‐IM group (Figure 3B,C).

### 3.4 Effect of route of vaccine administration on induction of Th CSCs in spleen and lungs

**FIGURE 4:**
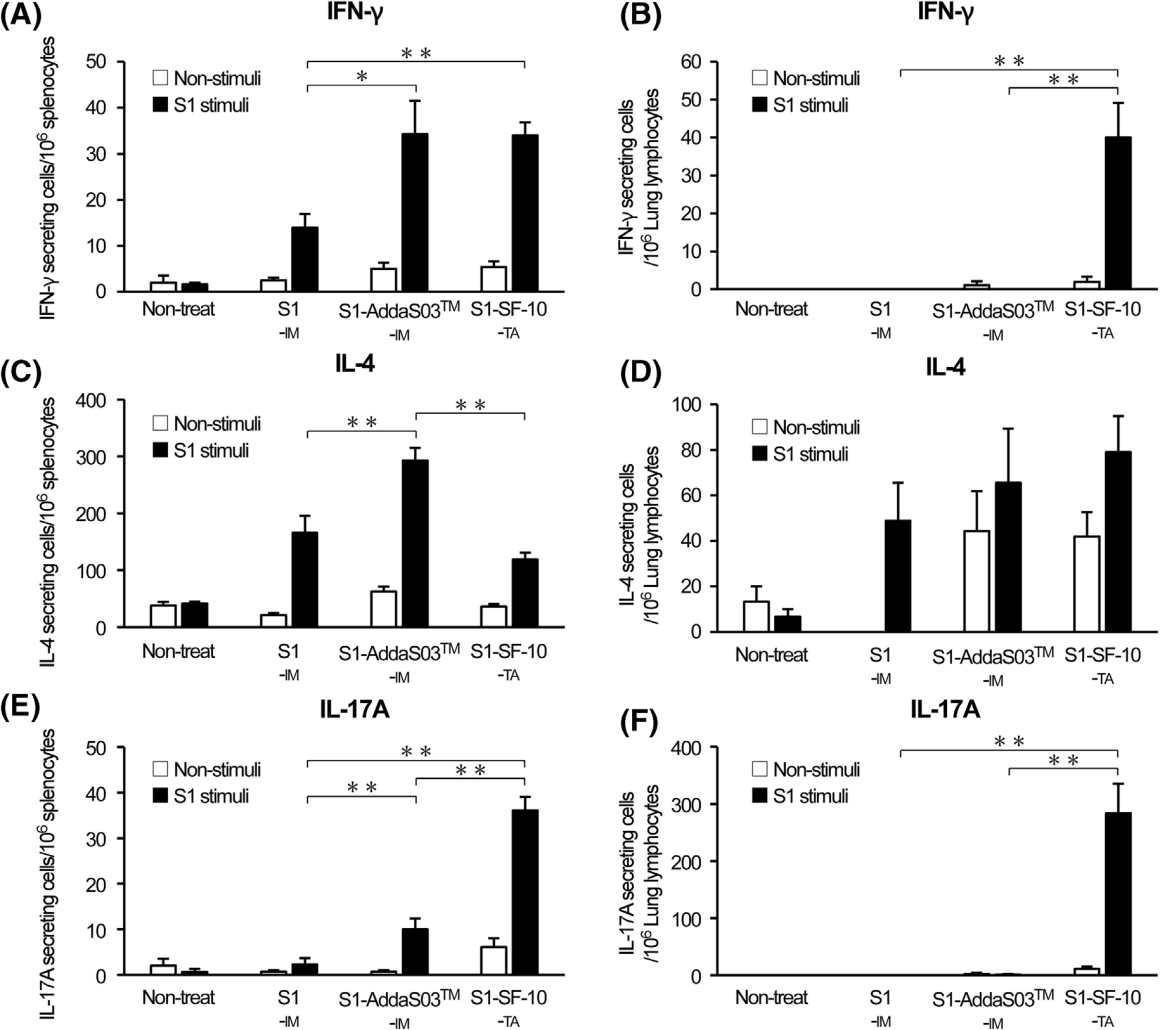
Detection of S1‐responsive CSCs in spleen and lung tissues. Mice were triple vaccinated by S1‐IM, S1‐AddaS03™‐IM, and S1‐SF‐10‐TA every 2 weeks. At 2 weeks after the last immunization, lymphocytes from the spleen and lungs of immunized mice (*n* = 10 mice per group) or non‐treated negative control mice (*n* = 3) were isolated, and then seeded and incubated with or without 20 μg/mL of S1 for 24 h. The IFN‐γ (A, B), IL‐4 (C, D), and IL‐17A (E, F) CSCs in lymphocytes of spleen (A, C, E) and lungs (B, D, F) were detected by ELISPOT. Data are mean ± SEM counts of non‐stimuli well (open bars) and S1‐stimuli well (closed bars) of CSC per 10^6^ cells (*n* = 10). Differences between groups were analyzed by the non‐parametric Mann–Whitney *U*‐test. *P < 0.05, **P < 0.01.

We reported previously that mucosal immunization of HAv‐SF‐10, induces antigen‐responsive Th1, Th2, and Th17 cytokines in the spleen.^19^ To analyze the T cell associated immunities against SARS‐CoV‐2 induced by S1‐SF‐10‐TA, we measured S1‐responsive Th CSCs in the spleen and lungs. Mice received triple vaccinations with S1‐IM, S1‐AddaS03™‐IM and S1‐SF‐10‐TA, and the splenocytes and lung lymphocytes of these mice were isolated at 2 weeks after the last vaccination, followed by incubation with or without S1 for 24 h. The S1‐responsive Th1 (IFN‐γ), Th2 (IL‐4), and Th17 (IL‐17A) CSCs were detected by ELISPOT assay (Figure 4). The levels of S1‐responsive IFN‐γ CSCs induced by S1‐AddaS03™‐IM and S1‐SF‐10‐TA in the spleen were comparable and higher than those induced by S1‐IM. In comparison, S1‐responsive IFN‐γ CSCs in lungs were only detected in the S1‐SF‐10‐TA group. Further analysis showed that the highest levels of S1‐responsive IL‐4 CSCs in the spleen were found in the S1‐AddaS03™‐IM group whereas those in the S1‐SF‐10‐TA group were slightly lower than those of the S1‐IM group. With regard to the lungs, similar levels of S1‐responsive IL‐4 CSCs were detected in the S1‐IM, S1‐AddaS03™‐IM, and S1‐SF‐10‐TA groups. The level of S1‐responsive IL‐17A CSCs in the spleen of the S1‐SF‐10‐TA group was the highest among the vaccination groups, and the S1‐responsive IL‐17A CSCs in the lungs were detected only in the S1‐SF‐10‐TA group.

## 4 DISCUSSION

In this study, we described new findings on TA route administration of mucosal adjuvant SF‐10 derived from PS for COVID‐19 vaccines. The main findings include (i) S1‐SF‐10‐TA vaccine induced S1‐specific IgG in serum to levels similar to those found with S1‐AddaS03™‐IM, together with induction of higher S‐IgA and IgG levels in the respiratory mucosa than S1‐AddaS03™‐IM, which plays an important role in protection against SARS‐CoV‐2 infection. (ii) S1‐SF‐10‐TA induced S1‐specific IgG and IgA ASCs and S1‐responsive IFN‐γ, IL‐4, and IL‐17A CSCs in both the spleen and lungs.

Respiratory mucosal immunity plays an important front‐line role against respiratory viral infections. Especially, S‐IgA in the respiratory mucosa protects against invasion of viruses into mucosal cells, resulting in prevention of infection and transmission of pathogens to other individuals.^12^, ^25^ Our results demonstrated that S1‐SF‐10‐TA vaccine induced S1‐specific IgG and IgA ASCs in the spleen and lungs (Figure 3) and S1‐specific antibodies in the serum and BALF (Figures 1 and 2A–D), which efficiently inhibited S1/ACE2 binding (Figure 2E–H). The S1‐AddaS03™‐IM induced S1‐specific IgG in the serum and BALF (Figure 2A,B) but not S1‐specific IgA in serum and BALF (Figure 2C,D), and S1‐specific IgA ASCs in the spleen and lungs (Figure 3B,D). The antibodies‐related inhibitory effects of S1/ACE2 binding in BALF induced by S1‐AddaS03™‐IM were also weaker than TA route application of S1‐SF‐10‐TA (Figure 2F,H).

In addition, the role of mucosal IgG induced by S1‐SF‐10‐TA cannot be ignored; S1‐SF‐10‐TA induced significantly higher BALF IgG levels compared with S1‐AddaS03™‐IM regardless of the time of administration (Figure 2B). Based on the above results, our findings suggest that vaccination with S1‐SF‐10‐TA provides efficient protection against respiratory infection.

In addition to protective humoral immunity, T cell‐mediated immunity is important for protection of SARS‐CoV‐2 infection. It was reported recently that SARS‐CoV‐2 antigen responsive IFN‐γ CSCs in blood plays an important role in the early phase of SARS‐CoV‐2 infection and acceleration of virus clearance.^26^ Also, IFN‐γ producing lung tissue‐resident memory T cells (TRMs) conduct immune surveillance for pathogens that could invade the tissues and robustly protect against site‐specific infection by viruses, including SARS‐CoV‐2 in the respiratory tract, thus preventing infection.^27^ S1‐AddaS03™‐IM induced S1‐responsive IFN‐γ CSCs in the spleen but not in the lungs. In contrast, S1‐SF‐10‐TA induced S1‐responsive IFN‐γ CSCs in not only the spleen but also in the lungs. Additionally, S1‐SF‐10‐TA vaccine induced S1‐responsive granzyme β expression in splenic CD8^+^ T cells, a marker of cellular immunity (Figure S2). Based on this likely scenario, we suggest that the use of TA administration for S1‐SF‐10 vaccine delivery offers the advantage of preventing respiratory infection, such as SARS‐CoV‐2, better than intramuscularly delivered vaccine.

Although S1‐SF‐10‐TA induced significantly higher splenic S1‐responsive IL‐17A CSCs relative to S1‐AddaS03™‐IM, splenic S1‐responsive IL‐4 CSCs induced by S1‐SF‐10‐TA were lower than those by S1‐AddaS03™‐IM (Figure 4E). These results indicate that S1‐SF‐10‐TA tends to induce mild Th2 type immunity and marked Th17 type immunity, compared with intramuscularly injected vaccine. Since IL‐17A is secreted by Th17 and involved in production and migration to mucosal sites of IgA,^28^, ^29^ these results imply that IL‐17A induced by S1‐SF‐10‐TA vaccine is mediated at least in part by marked IgA induction in serum and respiratory tract mucosa. Although the functional properties of vaccine‐induced Th17 TRM in the lungs remain to be examined in detail, Th17 TRM could mediate the induction of respiratory IgA following vaccination with S1‐SF‐10‐TA.^28^, ^29^ On the other hand, others indicated that Th17 induced by SARS‐CoV‐2 infection can induce a cytokine storm.^30^, ^31^ Since there are two types of Th17, the first is non‐pathogenic Th17, which produces IL‐10, has anti‐inflammatory activity, while the second is the pathogenic Th17, which does not induce IL‐10,^31^, ^32^ further studies are needed to determine whether lung Th17 induced by S1‐SF‐10‐TA immunization are non‐pathogenic type capable of inducing IL‐17A and IL‐10 to protect against SARS‐CoV‐2 infection.

Our study has certain limitations. We investigated protective immunity against SARS‐CoV‐2 infection using by S1/ACE2 BI assay in vitro. To confirm the protective immunity induced by mucosal administration of S1‐SF‐10 against mortality, severity, and transmission by SARS‐CoV‐2 infection in vivo, we need to conduct protection experiments in SARS‐CoV‐2‐infected animals.

In conclusion, we have demonstrated in the present study that SF‐10, which mimics human PS, is a suitable and effective mucosal adjuvant for COVID‐19 vaccines and can be used for TA delivery of vaccines to initiate rapid and potent local respiratory mucosal and systemic immunity. TA route administration of COVID‐19 vaccines using SF‐10 are expected to provide effective protection against respiratory infection of SARS‐CoV‐2.

## AUTHOR CONTRIBUTIONS

**Takashi Kimoto:** Data curation; formal analysis; funding acquisition; investigation; methodology; project administration; writing ‐ original draft; writing ‐ review and editing. **Satoko Sakai:** Data curation; formal analysis; investigation; methodology; validation; visualization. **Keiko Kameda:** Formal analysis; investigation; methodology; validation. **Ryoko Morita:** Formal analysis; methodology. **Etsuhisa Takahashi:** Formal analysis; investigation; methodology. **Yasuo Shinohara:** Investigation; methodology; project administration; validation. **Hiroshi Kido:** Conceptualization; data curation; funding acquisition; project administration; supervision; writing ‐ original draft; writing ‐ review and editing.

## CONFLICT OF INTEREST STATEMENT

The authors declare no conflict of interest.

## ETHICS STATEMENT

All mice used in this study were treated according to the Guide for the Care and Use of Laboratory Animals (NIH Publication No. 85‐23, 1996), and the study was approved by the Animal Care Committee of Tokushima University (#T2020‐65).

### PEER REVIEW

The peer review history for this article is available at https://publons.com/publon/10.1111/irv.13119.

## Supporting information


**Figure S1.** Changes in vaccine distribution in the airway and antibody induction efficacy with increasing doses of trans‐airway (TA) vaccine.
**Figure S2** Detection of Graβ‐producing CD8^+^ T cells in splenocytes.

## Data Availability

The data presented in this study are available in the supporting information of this article.

## References

[1] World Health Organization . Coronavirus disease (COVID‐19) dashboard. Accessed February 13, 2023. https://covid19.who.int

[2] Polack FP , Thomas SJ , Kitchin N , et al. Safety and efficacy of the BNT162b2 mRNA Covid‐19 vaccine. N Engl J Med. 2020;383(27):2603‐2615. doi:10.1056/NEJMoa2034577 33301246 PMC7745181

[3] Baden LR , el Sahly HM , Essink B , et al. Efficacy and safety of the mRNA‐1273 SARS‐CoV‐2 vaccine. N Engl J Med. 2021;384(5):403‐416. doi:10.1056/NEJMoa2035389 33378609 PMC7787219

[4] Voysey M , Clemens SAC , Madhi SA , et al. Safety and efficacy of the ChAdOx1 nCoV‐19 vaccine (AZD1222) against SARS‐CoV‐2: an interim analysis of four randomised controlled trials in Brazil, South Africa, and the UK. Lancet. 2021;397(10269):99‐111. doi:10.1016/S0140-6736(20)32661-1 33306989 PMC7723445

[5] Andrews N , Tessier E , Stowe J , et al. Duration of protection against mild and severe disease by Covid‐19 vaccines. N Engl J Med. 2022;386(4):340‐350. doi:10.1056/NEJMoa2115481 35021002 PMC8781262

[6] Holmgren J , Czerkinsky C . Mucosal immunity and vaccines. Nat Med. 2005;11(4 Suppl):S45‐S53. doi:10.1038/nm1213 15812489

[7] Ketas TJ , Chaturbhuj D , Portillo VMC , et al. Antibody responses to SARS‐CoV‐2 mRNA vaccines are detectable in saliva. Pathog Immun. 2021;6(1):116‐134. doi:10.1101/2021.03.11.434841 34136730 PMC8201795

[8] Chan RWY , Liu S , Cheung JY , et al. The mucosal and serological immune responses to the novel coronavirus (SARS‐CoV‐2) vaccines. Front Immunol. 2021;12:744887. doi:10.3389/fimmu.2021.744887 34712232 PMC8547269

[9] Azzi L , Dalla Gasperina D , Veronesi G , et al. Mucosal immune response in BNT162b2 COVID‐19 vaccine recipients. EBioMedicine. 2022;75:103788. doi:10.1016/j.ebiom.2021.103788 34954658 PMC8718969

[10] Davis SS . Nasal vaccines. Adv Drug Deliv Rev. 2001;51(1‐3):21‐42. doi:10.1016/s0169-409x(01)00162-4 11516777

[11] Knowles MR , Boucher RC . Mucus clearance as a primary innate defense mechanism for mammalian airways. J Clin Invest. 2002;109(5):571‐577. doi:10.1172/JCI15217 11877463 PMC150901

[12] Correa VA , Portilho AI , De Gaspari E . Vaccines, adjuvants and key factors for mucosal immune response. Immunology. 2022;167(2):124‐138. doi:10.1111/imm.13526 35751397

[13] Grohskopf LA , Alyanak E , Ferdinands JM , et al. Prevention and control of seasonal influenza with vaccines: recommendations of the advisory committee on immunization practices, United States, 2021‐22 influenza season. MMWR Recomm Rep. 2021;70(5):1‐28. doi:10.15585/mmwr.rr7005a1 PMC8407757 34448800

[14] Kimoto T . Development of a safe and effective novel synthetic mucosal adjuvant SF‐10 derived from physiological metabolic pathways and function of human pulmonary surfactant. Vaccine. 2022;40(3):544‐553. doi:10.1016/j.vaccine.2021.11.030 34887132

[15] Mizuno D , Ide‐Kurihara M , Ichinomiya T , Kubo I , Kido H . Modified pulmonary surfactant is a potent adjuvant that stimulates the mucosal IgA production in response to the influenza virus antigen. J Immunol. 2006;176(2):1122‐1130. doi:10.4049/jimmunol.176.2.1122 16394001

[16] Nishino M , Mizuno D , Kimoto T , et al. Influenza vaccine with Surfacten, a modified pulmonary surfactant, induces systemic and mucosal immune responses without side effects in minipigs. Vaccine. 2009;27(41):5620‐5627. doi:10.1016/j.vaccine.2009.07.024 19647064

[17] Mizuno D , Kimoto T , Takei T , et al. Surfactant protein C is an essential constituent for mucosal adjuvanticity of Surfacten, acting as an antigen delivery vehicle and inducing both local and systemic immunity. Vaccine. 2011;29(33):5368‐5378. doi:10.1016/j.vaccine.2011.05.090 21669246

[18] Kimoto T , Mizuno D , Takei T , et al. Intranasal influenza vaccination using a new synthetic mucosal adjuvant SF‐10: induction of potent local and systemic immunity with balanced Th1 and Th2 responses. Influenza Other Respi Viruses. 2013;7(6):1218‐1226. doi:10.1111/irv.12124 PMC3933764 23710832

[19] Kimoto T , Kim H , Sakai S , Takahashi E , Kido H . Oral vaccination with influenza hemagglutinin combined with human pulmonary surfactant‐mimicking synthetic adjuvant SF‐10 induces efficient local and systemic immunity compared with nasal and subcutaneous vaccination and provides protective immunity in mice. Vaccine. 2019;37(4):612‐622. doi:10.1016/j.vaccine.2018.12.002 30553569

[20] Mizuno D , Kimoto T , Sakai S , Takahashi E , Kim H , Kido H . Induction of systemic and mucosal immunity and maintenance of its memory against influenza a virus by nasal vaccination using a new mucosal adjuvant SF‐10 derived from pulmonary surfactant in young cynomolgus monkeys. Vaccine. 2016;34(16):1881‐1888. doi:10.1016/j.vaccine.2016.02.061 26954466

[21] Kim H , Kimoto T , Sakai S , Takahashi E , Kido H . Adjuvanting influenza hemagglutinin vaccine with a human pulmonary surfactant‐mimicking synthetic compound SF‐10 induces local and systemic cell‐mediated immunity in mice. PLoS ONE. 2018;13(1):e0191133. doi:10.1371/journal.pone.0191133 29370185 PMC5784949

[22] Arunachalam PS , Walls AC , Golden N , et al. Adjuvanting a subunit COVID‐19 vaccine to induce protective immunity. Nature. 2021;594(7862):253‐258. doi:10.1038/s41586-021-03530-2 33873199

[23] Goepfert PA , Fu B , Chabanon A‐L , et al. Safety and immunogenicity of SARS‐CoV‐2 recombinant protein vaccine formulations in healthy adults: interim results of a randomised, placebo‐controlled, phase 1–2, dose‐ranging study. Lancet Infect Dis. 2021;21(9):1257‐1270. doi:10.1016/S1473-3099(21)00147-X 33887209 PMC8055206

[24] Wu Y , Wang F , Shen C , et al. A noncompeting pair of human neutralizing antibodies block COVID‐19 virus binding to its receptor ACE2. Science. 2020;368(6496):1274‐1278. doi:10.1126/science.abc2241 32404477 PMC7223722

[25] Sterlin D , Mathian A , Miyara M , et al. IgA dominates the early neutralizing antibody response to SARS‐CoV‐2. Sci Transl Med. 2021;13(577):eabd2223. doi:10.1126/scitranslmed.abd2223 33288662 PMC7857408

[26] Tan AT , Linster M , Tan CW , et al. Early induction of functional SARS‐CoV‐2‐specific T cells associates with rapid viral clearance and mild disease in COVID‐19 patients. Cell Rep. 2021;34(6):108728. doi:10.1016/j.celrep.2021.108728 33516277 PMC7826084

[27] Lei H , Alu A , Yang J , et al. Intranasal administration of a recombinant RBD vaccine induces long‐term immunity against omicron‐included SARS‐CoV‐2 variants. Signal Transduct Target Ther. 2022;7(1):159. doi:10.1038/s41392-022-01002-1 35581200 PMC9112270

[28] Hirota K , Turner JE , Villa M , et al. Plasticity of Th17 cells in Peyer's patches is responsible for the induction of T cell‐dependent IgA responses. Nat Immunol. 2013;14(4):372‐379. doi:10.1038/ni.2552 23475182 PMC3672955

[29] Cao AT , Yao S , Gong B , Elson CO , Cong Y . Th17 cells upregulate polymeric Ig receptor and intestinal IgA and contribute to intestinal homeostasis. J Immunol. 2012;189(9):4666‐4673. doi:10.4049/jimmunol.1200955 22993206 PMC3478497

[30] De Biasi S , Meschiari M , Gibellini L , et al. Marked T cell activation, senescence, exhaustion and skewing towards TH17 in patients with COVID‐19 pneumonia. Nat Commun. 2020;11(1):3434. doi:10.1038/s41467-020-17292-4 32632085 PMC7338513

[31] Martonik D , Parfieniuk‐Kowerda A , Rogalska M , Flisiak R . The role of Th17 response in COVID‐19. Cell. 2021;10(6):1550. doi:10.3390/cells10061550 PMC8235311 34205262

[32] McGeachy MJ , Bak‐Jensen KS , Chen Y , et al. TGF‐beta and IL‐6 drive the production of IL‐17 and IL‐10 by T cells and restrain T(H)‐17 cell‐mediated pathology. Nat Immunol. 2007;8(12):1390‐1397. doi:10.1038/ni1539 17994024

