# Supplementary material for: Induction of systemic, mucosal, and cellular immunity against SARS‐CoV‐2 in mice vaccinated by trans‐airway with a S1 protein combined with a pulmonary surfactant‐derived adjuvant SF‐10
Source: Influenza Other Respir Viruses. 2023 Mar 9;17(3):e13119. doi: 10.1111/irv.13119 (PMC9996429; doi:10.1111/irv.13119)
Supplement: Supplementary file 1 — Figure S1. Changes in vaccine distribution in the airway and antibody induction efficacy with increasing doses of trans‐airway (TA) vaccine. Figure S2 Detection of Graβ‐producing CD8+ T cells in splenocytes. [file IRV-17-e13119-s001.docx]

**
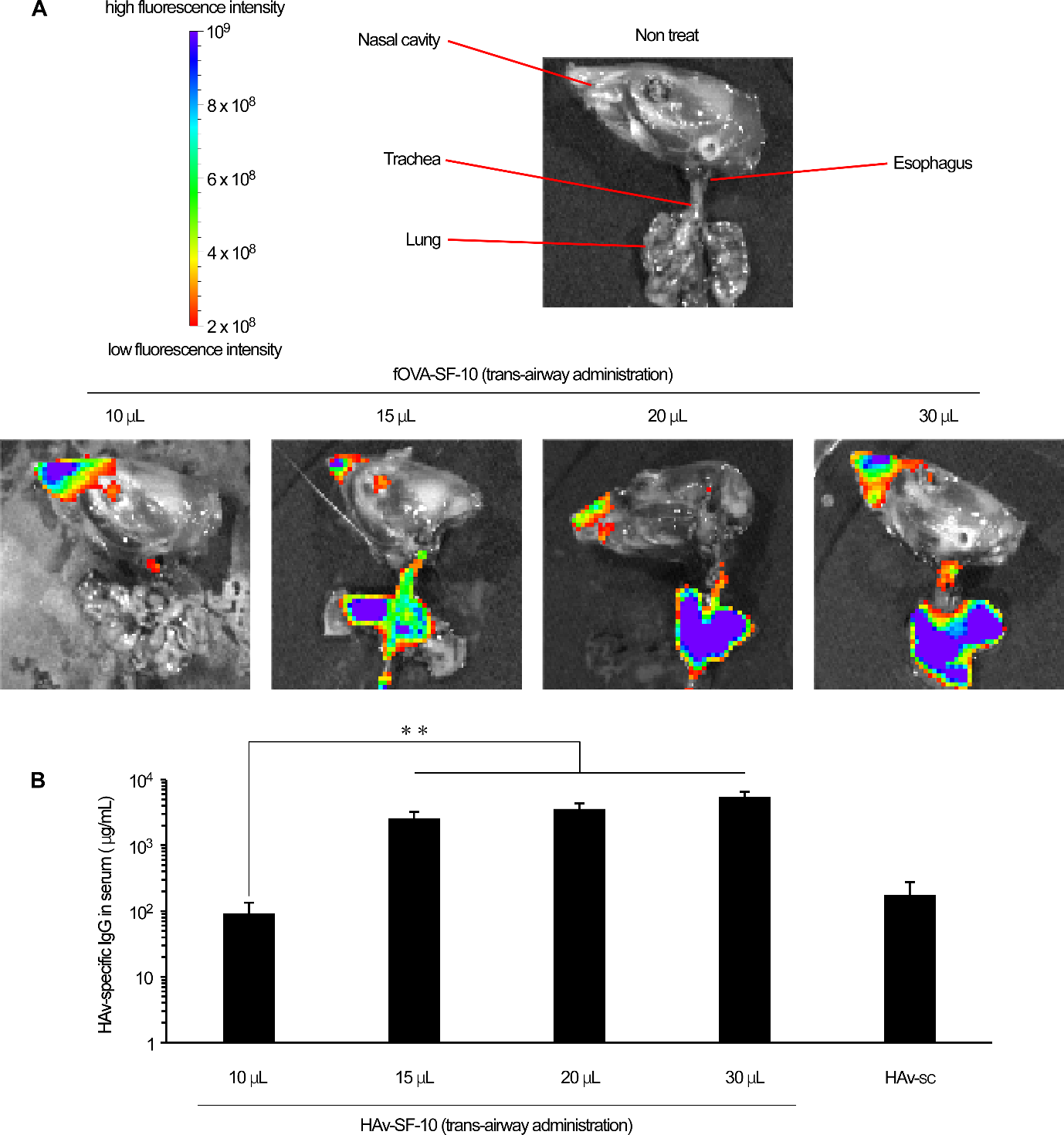
**

**SUPPLEMENTARY FIGURE 1 Changes in vaccine distribution in the airway**

**and antibody induction efficacy with increasing doses of trans-airway (TA) vaccine.**

Mice were inoculated from nasal cavity with increasing doses at 10 to 30 μL of TA vaccine, which contains 5 μg Alexa647-labeled ovalbumin (fOVA, Thermo Fisher Scientific, Middletown, VA) and 50 μg SF-10 (phospholipids) in 0.1% CVP. Immediately after administration, head, trachea, lungs and esophagus were isolated to analyze fOVA distribution by in vivo imaging system (IVIS Spectrum, Perkin Elmer, CA).

(A) Vaccine distribution in the airways with increasing doses of TA vaccine.

At a dose of 10 μL TA vaccine, the vaccine was distributed only in the nasal cavity, but when this dose was increased to 15-30 μL, the vaccine was distributed from the nasal cavity to the lower respiratory tract.

(B) Increase in efficacy of HAv specific IgG induction in serum with increase in TA vaccine dosage.

Although the amount of HAv antigen (A/California/7/2009(H1N1), Kitasato Daiichi Sankyo Vaccine Co., Tokyo, Japan) at 1 μg with 10 μg SF-10 in 0.1% CVP administrated at days 0 and 14 was identical between the 10 and 15-30 µL doses of TA vaccine, induced HAv-specific IgG in serum analyzed at 2 weeks after the last immunization was increased dramatically at several dozen-fold along with increased TA vaccine distribution. As a control group, mice were subcutaneously injected with 1 μg HAv in 100 μL saline [HAv-sc] with the same dosing schedule.^18-21^ Data are geometric mean ± SEM of 6 mice. Differences between groups were analyzed by the non-parametric Mann–Whitney U-test. ^**^*P* < 0.01.

**
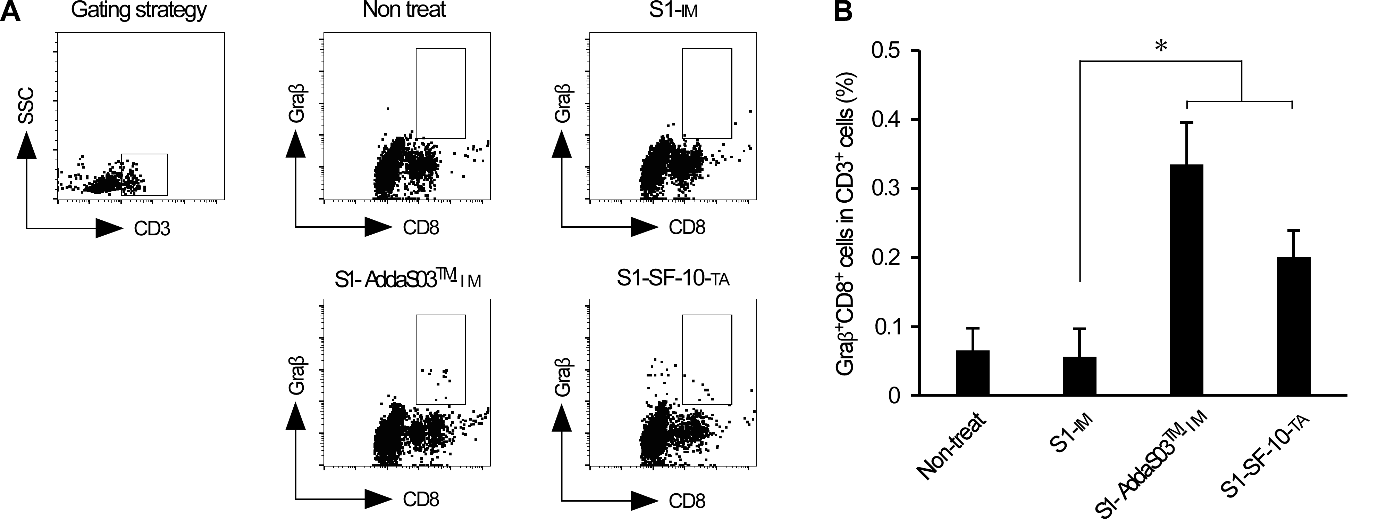
**

**SUPPLEMENTARY FIGURE 2 Detection of Graβ-producing CD8^+^ T cells in splenocytes.**

Splenocytes of mice immunized three times with 10 μg of S1-IM, S1-AddaS03^TM^ and S1-SF-10-TA were isolated at 2 weeks after the last immunization. For detection of Granzyme β (Graβ)-producing CD8^+^ T cells, the splenocytes were incubated in cRPMI containing 20 μg/mL S1 protein for 3 days, followed by further incubation for 6 h in the presence of BD Golgiplug Protein Transport Inhibitor (Containing Brefeldin A) (BD Biosciences, Franklin Lakes, NJ).^21^ Next, the cells were stained with anti-mouse CD3 Ab (BioLegend) and anti-mouse CD8α Ab (BioLegend), fixed and permeabilized by Transcription Factor Buffer Set (BD Biosciences), and stained with anti-mouse Graβ Ab (BioLegend). Flowcytometry was performed using CytoFLEX (Beckman Coulter, CA) and Flowjo software (BD Biosciences). (A) Graβ producing cells CD8^+^ T cells on gated CD3^+^ T cells were analyzed by flowcytometry. (B) Data of gated Graβ^+^CD8^+^ T cells population are represented as the mean ± SEM of each immunization group (n = 6 mice per group) and negative control untreated group (n = 3). Differences between groups were analyzed by the non-parametric Mann–Whitney U-test. ^*^*P* < 0.05.
